# How many parathyroid glands can be identified during thyroidectomy? Evidence-based data for medical experts

**DOI:** 10.1007/s10353-017-0502-0

**Published:** 2017-12-13

**Authors:** Elisabeth Gschwandtner, Rudolf Seemann, Claudia Bures, Lejla Preldzic, Eduard Szucsik, Michael Hermann

**Affiliations:** 1 Second Department of Surgery “Kaiserin Elisabeth”, Krankenanstalt Rudolfstiftung, Juchgasse 25, 1030 Vienna, Austria; 2 Department of Cranio‑, Maxillofacial and Oral Surgery, Medical University Vienna, Vienna, Austria

**Keywords:** Hypoparathyroidism, Thyroid surgery, Parathyroid glands, Quality control, Autotransplantation

## Abstract

**Background:**

The purpose of this study is to provide guidance for medical experts regarding malpractice claims on permanent hypoparathyroidism by analyzing the number of parathyroid glands (PGs) identified during thyroidectomy and the clinical outcome.

**Methods:**

Parathyroid findings were documented in a standardized protocol for 357 patients undergoing thyroidectomy and treated by a single specialized surgeon. The resected thyroid was routinely dissected for accidentally removed PGs with consecutive autotransplantation and the pathological report also described unintentionally resected PGs. Follow-up was performed for 6 months.

**Results:**

The mean number of identified PGs was 2.28. No PGs were found in 20 (5.6%), one in 56 (15.7%), two in 126 (35.3%), three in 114 (31.9%), and four in 41 (11.5%) cases. One patient (0.28%) had manifest permanent hypoparathyroidism, while ten patients (2.8%) had latent permanent hypoparathyroidism (hypocalcemia and normoparathyroidism). The risk factors identified for postoperative hypoparathyroidism were an increasing number of visualized PGs, autotransplantation, central neck dissection, and PGs in the histopathological work-up. For permanent hypoparathyroidism, PGs in the histology examination and neck dissection were significant, but the number of identified PGs was not.

**Conclusion:**

Even an experienced surgeon is not always able to find all four PGs during thyroidectomy and occasionally identifies none. Rather than focusing on identifying a minimum number of PGs, it is more important not to miss them in risky positions. A documented awareness of PGs, i. e., knowledge of variable parathyroid positions and their saving, is a prerequisite for surgical quality and to protect surgeons from claims.

## Introduction

Postoperative hypoparathyroidism is an ongoing and frequently underestimated complication in thyroid surgery [1]. In the literature, transient hypocalcemia can be found in 1–64.4% of cases [2–5] and permanent complications in 0.5–17.4% of cases [6–12]. Long-term parathyroid insufficiency can be divided into two types: *latent permanent* hypoparathyroidism, characterized by hypocalcemia with or without symptoms and parathyroid levels in the (lower) normal range; and *manifest permanent *hypoparathyroidism, with decreased or no longer detectable parathyroid hormone (PTH) levels [13, 14]. Permanent hypoparathyroidism may result from accidental removal or vascular damage of the parathyroid glands (PGs).

Not all the PGs, however, can be visualized during thyroid surgery owing to their variable anatomic localization; indeed, an ectopic PG can be found in up to 16% of cases [15, 16]. This can also be seen from surgery of primary hyperparathyroidism as PGs may be ectopic or at a substantial distance from the thyroid [16–19] such that special efforts and extensive surgical procedures have to be undertaken to access the ectopic parathyroid adenoma. In thyroidectomy, an ectopic PG would be located in a safe position and thus not be at risk during removal of the thyroid gland.

State-of-the-art guidelines for the identification and preservation of PGs are not currently available and the opinions expressed in the literature are divergent. Some authors claim that a minimum number of PGs have to be visualized and saved: two according to Thomusch et al. [20], three according to Pattou et al. [6]. Others recognize the danger of active search and dissection as possible causes of devascularization [21–23]. The purpose of this study was to determine how many PGs can be identified and saved in situ during thyroid surgery, and also how many PGs can be found in the removed specimen via dissection by the surgeon or detected by the pathologist in his or her work-up procedure, if a subtle and careful microsurgical dissection technique is performed in thyroid surgery. The rate of hypoparathyroidism and risk factors for hypocalcemia after total thyroidectomy were analyzed.

This article should also serve as specific guidance for medical experts who have to evaluate a claim of malpractice concerning permanent hypoparathyroidism in cases where not all PGs could be visualized during thyroid surgery. They currently rely on guidelines or recommendations from the literature to form their verdict, but very few publications deal directly with this delicate issue [24].

## Methods

After approval by the ethical review board, the prospective institutional surgical database was the starting point for this retrospective study, which was conducted to determine the number of visualized PGs during thyroidectomy as well as the confounding factors [25]. A total of 357 patients who underwent near-total or total thyroidectomy between January 2005 and December 2012 were included. We have previously published details of the protocol and the data collection and processing [26–29].

The inclusion criteria were preoperative normocalcemia (2.1–2.7 mmol/l) and normoparathyroidism (11.0–67.0 pg/ml), complete pre- and postoperative serum levels of PTH and calcium, including long-term follow-up for at least 6 months as well as the documentation of hypocalcemic symptoms. We did not routinely place patients on calcium or vitamin D supplementation preoperatively. All operations were performed by a single surgeon who is specialized in thyroid and parathyroid surgery. A microsurgical technique with magnifying spectacles was used in all surgeries to aid the identification and dissection of the PGs. It is important to note that PGs were preserved if possible. If they were identified in an exposed position close to the resection line, maximum effort was made to save them with their blood supply. If not otherwise possible, a small thyroid remnant (i. e., near-total thyroidectomy) was left in order to save the PG in situ. The surgeon did not, however, explicitly search for PGs if they could not be identified within the surgical field. All resected specimens were carefully observed for accidentally resected PGs by the surgeon and this was also described in the surgical report. If preservation of a PG was not possible or if a PG was located on the resected specimen, parathyroid autotransplantation was performed. Autotransplantation was usually done by inserting the fragmented PG in the ipsilateral sternocleidomastoid muscle. Furthermore, the pathologists were also instructed to include accidentally resected PGs in their pathological report. The analysis of the postoperative serum levels of PTH and calcium were routinely performed after every thyroid surgery on the first postoperative day. As part of the quality assurance, all patients with complications (i. e., hypoparathyroidism, recurrent laryngeal nerve palsy) were given appointments for routinely performed follow-up examinations in our clinic [4, 13]. These routine appointments gave us the opportunity to undertake clinical and laboratory follow-up examinations at the institution and they take place 2 weeks, 3 months, and 6 months after surgery and every 6 months thereafter.

The inpatient and outpatient data collected included gender, age, surgical indication, surgical technique of right and left thyroid lobe (near-total or total procedure) without or with central nodal dissection, number of preserved PGs, number of autotransplanted PGs (due to impaired or lack of blood supply or because of late identification in the resected specimen during thyroid surgery), postoperatively histopathologically verified PGs through the study of the pathological reports, non-identified PGs during surgery, pre- and postoperative levels of PTH and calcium, along with follow-up data for at least 6 months.

We defined transient or postoperative hypoparathyroidism as subnormal PTH and/or calcium levels with or without the need of supplementation for a maximum duration of 6 months. Permanent hypoparathyroidism was further divided into *manifest permanent* and *latent permanent*. Manifest permanent hypoparathyroidism was defined as subnormal PTH and calcium levels with the need for supplementation, and latent permanent hypoparathyroidism or parathyroid insufficiency was defined as PTH levels in the lower normal range and hypocalcemia with or without symptoms and with or without the need for calcium or vitamin D substitution for over 6 months.

### Statistical analysis

Logistic regression models of transient and permanent hypoparathyroidism were tested with each factor in univariate models and in a multivariate approach comprising all four factors (preserved PGs, autotransplantation, confirmed removal during pathologic work-up, and central neck dissection). Odds ratios (OR) and 95% confidence intervals (CI) of odds ratios were computed. A *p *value of the likelihood ratio test (comparing the model with factor to a model without the factor) less than 0.05 was considered to be significant. All statistical tests were performed using the open source statistical programming environment R—version 3.1.1 (http://www.r-project.org).

## Results

A total of 357 patients (288 women: 55.6 ± 14.2 years old, range: 18–89 years; 69 men: 59.0 ± 12.0 years old, range: 35–83 years) were included in this study. The histological pathology of the thyroid gland was a nodular goiter in 262 (73.4%) patients, 50 (14.0%) had Grave’s disease, six (1.9%) had Hashimoto thyroiditis, and 39 (10.9%) patients had thyroid cancer (four follicular, three medullary, 20 papillary carcinomas, 12 papillary microcarcinomas). Total thyroidectomy was performed on 276 patients, near-total thyroidectomy on 22 patients, and a combination of lobectomy and near-total lobectomy on 59 patients.

### Incidence of hypoparathyroidism

A total of 105 (29.4%) patients showed pathological serum levels of either PTH or calcium or both at discharge on the second postoperative day. In the long-term follow-up (more than 1 year), one patient (0.28%) suffered from manifest permanent hypoparathyroidism, i. e., subnormal PTH and calcium levels with the need for supplementation, but ten patients (2.8%) had latent permanent hypoparathyroidism or parathyroid insufficiency, i. e., PTH levels in the lower normal range and hypocalcemia with or without symptoms and with or without the need for calcium or vitamin D substitution. Consequently, during long-term follow-up, 346 (96.9%) patients had normal PTH and calcium levels without symptoms of hypoparathyroidism.

In the one male patient with manifest permanent hypoparathyroidism, no PGs could be identified in situ during surgery, but one PG was autotransplanted. This PG was found after removal of the first thyroid lobe and the subsequent microsurgical search for the resected thyroid specimen where it was identified in the thyroid capsule and then autotransplanted into the ipsilateral sternocleidomastoid muscle. Another PG was found during histopathological work-up invaginated in the thyroid gland and missed by the surgeon. This patient did not have central nodal dissection.

As depicted in Table 1, ten patients (female:male = 9:1) were diagnosed with latent permanent hypoparathyroidism. Four of the ten patients with latent hypoparathyroidism had an autotransplantation. Five patients had a lymphadenectomy. Two PGs were found during the histopathological work-up. In the group with latent permanent hypoparathyroidism, the mean number of identified PGs was 2.2 (range: 1–3).

**Table 1:** Patients with latent permanent and manifest hypoparathyroidism

Patient	Hypoparathyroidism	Lymphadenectomy	PG in pathology	PG visualized and saved during surgery	PG autotransplantation
1	Manifest permanent	No	1	0	1
2	Latent permanent	Yes	1	1	1
3	Latent permanent	Yes	1	2	0
4	Latent permanent	Yes	0	2	0
5	Latent permanent	Yes	0	2	0
6	Latent permanent	Yes	0	2	1
7	Latent permanent	No	0	2	0
8	Latent permanent	No	0	3	0
9	Latent permanent	No	0	2	0
10	Latent permanent	No	0	3	1
11	Latent permanent	No	0	3	1

*PG *parathyroid gland

### Identified parathyroid glands

**Fig. 1:**
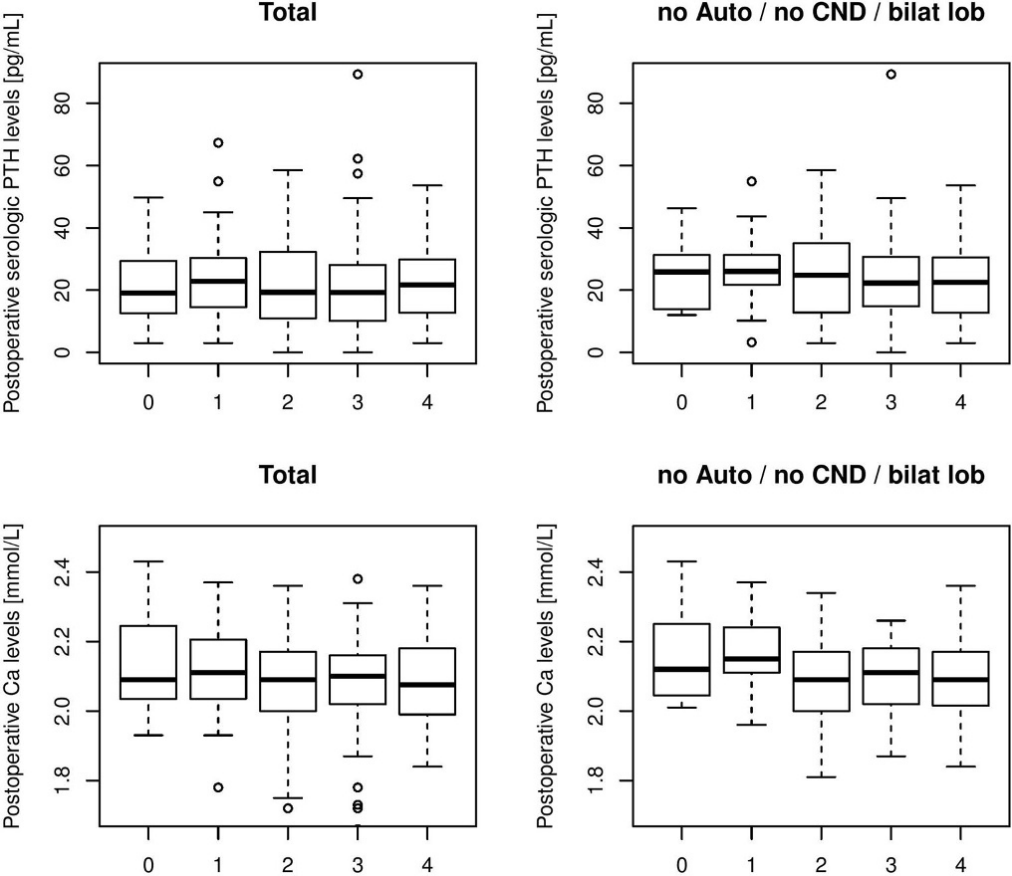
Postoperative parathyroid hormone (*PTH*) and serum calcium (*Ca*) levels on the first postoperative day depending on the number of identified parathyroid glands with and without autotransplantation (*Auto*) and central nodal dissection (*CND*). *bilat lob* Bilateral lobectomy

On average, 2.28 PGs were identified during thyroidectomy. No PG was found in situ in 20 (5.6%), one in 56 (15.7%), two in 126 (35.3%), three in 114 (31.9%), and four in 41 (11.5%) cases. With an increasing number of visualized PGs, we found a significantly higher ratio of postoperative hypoparathyroidism (none: 20%/*n* = 4; one: 17.9%/*n* = 10; two: 30.2%/*n* = 38; three: 36.0%/*n* = 41; four: 31.7%/*n* = 13; logistic regression model: OR = 1.28, 95% CI: 1.02–1.6; *p*_LR_ = 0.030; see Fig. 1). We also observed a trend toward a decreasing ratio of permanent hypoparathyroidism (none: 5.0%/*n* = 1; one: 1.8%/*n* = 1; two: 4.8%/*n* = 6; three: 2.6%/*n* = 3; four: 0.0%/*n* = 0) but this did not reach significance (logistic regression model: OR = 0.77, 95% CI: 0.44–1.36; *p*_LR_ = 0.369). In the multivariate models, transient hypoparathyroidism again reached significance (Tables 2 and 3).

**Table 2:** Logistic regression predicting postoperative hypoparathyroidism

Variables	Adjusted OR (95% CI)	*p* (likelihood ratio test)
Histologically verified parathyroid glands 1 vs. 0	3.05 (1.12–8.33)	0.029
In situ identified and preserved parathyroid glands	1.49 (1.16–1.92)	0.001
Parathyroid glands—autotransplantation	2.2 (1.31–3.7)	0.003
Central nodal dissection 1 vs. 0	2.6 (1.23–5.47)	0.013

*CI* confidence interval, *OR* odds ratio

The log-likelihood was −199.3069 in 357 patients, the Akaike information criterion was 408.6139

**Table 3:** Logistic regression predicting permanent hypoparathyroidism

Variables	Adjusted OR (95% CI)	*p* (likelihood ratio test)
Histologically verified parathyroid glands 1 vs. 0	3.42 (0.69–17.01)	0.155
In situ identified and preserved parathyroid glands	0.84 (0.44–1.61)	0.599
Parathyroid glands −autotransplantation	1.21 (0.35–4.13)	0.767
Central nodal dissection 1 vs. 0	4.57 (1.04–20.13)	0.052

*CI *confidence interval, *OR* odds ratio

The log-likelihood was −43.6197 in 357 patients, the Akaike information criterion was 97.2394

### Autotransplantation

In 272 (76.2%) patients, no PGs were autotransplanted. In 79 (22.1%) cases we autotransplanted one PG, and in six (1.7%) patients two. With an increasing number of autotransplanted PGs, we found a significantly higher ratio of postoperative hypoparathyroidism (none: 25.7%/*n* = 70; one: 39.2%/*n* = 31; two: 83.3%/*n* = 5; logistic regression model: OR = 2.19, 95% CI: 1.38–3.47; *p*_LR_ < 0.001). This trend was not confirmed regarding permanent hypoparathyroidism (none: 2.2%/*n* = 6; one: 6.3%/*n* = 5; two: 0.0%/*n* = 0) and did not reach significance (logistic regression model: OR = 2.07, 95% CI: 0.74–5.77; *p*_LR_ = 0.191). In the multivariate models, we only confirmed the significance of postoperative hypoparathyroidism (Tables 2 and 3).

### Parathyroid glands in histopathological work-up

In 20 (5.6%) patients, one PG was found during the histopathological work-up. We observed significantly more postoperative hypoparathyroidism (none: 27.9%/*n* = 94; one: 60.0%/*n* = 12) if one PG had been accidentally removed and found during histopathological work-up (logistic regression model: OR = 3.88, 95% CI: 1.54–9.79; *p*_LR_ = 0.004). Equivalently, significantly more cases of latent permanent or manifest permanent hypoparathyroidism (none: 2.4%/*n* = 8; one: 15.0%/*n* = 3) were found in the case of PG removal using univariate analysis (logistic regression model: OR = 7.26, 95% CI: 1.77–29.83; *p*_LR_ = 0.017). In the multivariate models, we could only confirm significance for postoperative hypoparathyroidism (Tables 2 and 3).

### Central nodal dissection

Thyroidectomy for benign thyroid pathologies was performed in 316 (88.5%) cases; malignant tumors were followed by central lymphadenectomy in 41 (11.5%) patients. Few patients had a prophylactic nodal dissection owing to a suspicious or unclear histological result in the frozen section examination. Significantly more postoperative hypoparathyroidism (no: 25.9%/*n* = 82, central neck: 58.5%/*n* = 24) was observed where central nodal dissection was performed (logistic regression model: OR = 4.03, 95% CI: 2.06–7.88; *p*_LR_ < 0.001). Consistently, central nodal dissection (no: 1.9%/*n* = 6, central nodal dissection: 12.2%/*n* = 5) was followed by a significantly higher risk of permanent latent hypoparathyroidism (logistic regression model: OR = 7.18, 95% CI: 2.09–24.7; *p*_LR_ = 0.004). In the multivariate models, only the significance of transient hypoparathyroidism was confirmed (Table 2); in the permanent hypoparathyroidism model, the significance level was almost reached (Table 3).

## Discussion

There is still a controversial discussion over how many PGs should be found during thyroidectomy to prevent hypoparathyroidism and whether or not surgeons should actively search for, dissect, and try to find unidentified PGs. Our study focused on the feasibility of PG identification in routine thyroid surgery in order to define standards that could be reflected in guidelines.

This question is also of great forensic relevance because medical experts often act on the assumption that four PGs have to be visualized during thyroidectomy to fulfil the standard of care. Our study shows that this goal is unattainable. It is crucial that decisions concerning malpractice claims are guided by evidence-based data and state-of-the-art guidelines. We should consider, for example, whether non-identification of PGs in thyroidectomy can be evaluated as malpractice in cases of permanent hypoparathyroidism. Our data demonstrate that, even for experienced surgeons, the preservation of all PGs is not always possible. This can also be explained by the highly variable location of PGs. Delattre et al. showed that in 4% of the patients all four PGs were located at a safe distance from the thyroid and were therefore not at risk of devascularization during thyroidectomy [18]. From surgery of hyperparathyroidism, we know that up to 16% [15, 16] of parathyroid adenomas are located in an ectopic position and are therefore protected in the thyroidectomy process.

Olson et al. showed that the risk for permanent hypoparathyroidism was significantly increased if fewer than three PGs were preserved [30]. Other authors have found that the localization of one or two PGs was required to avoid the complication [31–33]. In his recent publication, Sitges-Serra showed that the number of PGs identified was inversely correlated with postoperative, protracted, and permanent parathyroid insufficiency. When four, three, and one to two PGs could be saved in situ, permanent hypoparathyroidism occurred in 2.6%, 6.5%, and 16% of cases, respectively [34].

By contrast, Grimm et al. demonstrated in their multicenter study that the rate of permanent hypoparathyroidism was significantly lower if no PG was identified during thyroid surgery [35]. Similar results were found by Prazenica et al., who showed a higher rate of postoperative hypoparathyroidism with more identified PGs [36]. Sheahan et al. do not recommend a routine identification of all four PGs if a capsular dissection technique is being used [23]. Lang et al. reported that using an extracapsular technique in thyroidectomy and the identification of fewer PGs in their orthotopic localizations resulted in less temporary and permanent hypoparathyroidism and, furthermore, decreased the time to recovery from temporary hypoparathyroidism [37]. Other publications could not establish a correlation between the number of preserved PGs and symptomatic hypocalcemia [7, 38].

In our retrospective analysis, we found not a single PG in 20 (5.6%), one PG in 56 (15.7%), two PGs in 126 (35.3%), three PGs in 114 (31.9%), and four PGs in 41 (11.5%) cases. On average, 2.28 PGs could be preserved. Furthermore, our data show that the number of identified PGs initially had a significant negative effect on their secretory function, resulting in an increased number of postoperative hypoparathyroidism. In the long-term follow-up, however, the number of visualized PGs had positive, but nonsignificant, effects and by trend reduced the risk of permanent hypoparathyroidism.

International guidelines for the management of PGs in thyroid surgery are missing. In a study by Dralle et al., 28% of the malpractice claims in thyroid surgery involved hypoparathyroidism [24]. Generally, identification and preservation of the PGs are needed and the recording of their exact positions (in situ or autotransplanted) in the surgical report are necessary. It is not sufficient to state that no PG was visualized on the removed specimen [39]. Dralle et al. also showed that nine out of 12 malpractice cases resulted in defense verdicts [24]. This was mainly attributed to the dilemma regarding the lack of standard guidelines, which meant that the benefit of the doubt was applied frequently. Even without PG identification, most often due to fear of harming the blood supply of the PG, seven out of nine verdicts were defense verdicts. In general, it is desirable to identify and preserve all PGs in situ, but our study shows that it is not always possible. In some cases, none at all can be visualized. Consequently, the non-identification of PGs cannot be evaluated as malpractice if it is thoroughly documented in the surgical report that no PGs were found in the risky anatomical positions or within the resection line.

Our results also demonstrate that even endocrine experts can miss or misinterpret PGs. Histopathologically verified PGs, which were overlooked by the surgeon, had a greater impact on transient and permanent hypoparathyroidism. We found that patients in whom one PG was detected at the histopathological examination had a significantly increased risk for postoperative as well as permanent hypoparathyroidism. Unintended parathyroidectomy can occur in up to 22% of patients undergoing thyroid surgery [12, 40].

According to several studies, the extent of resection highly correlated with the outcome of hypoparathyroidism [8, 20, 41]. Testini et al. and Dubernard et al. demonstrated that hypoparathyroidism occurs more frequently after total thyroidectomy with central neck dissection or completion thyroidectomy [42, 43]. Similar findings were obtained in our study. Patients with central nodal dissection had a significantly higher risk of developing transient as well as permanent hypoparathyroidism.

The autotransplantation of PGs is the recommended surgical technique if a PG is devascularized or accidentally removed during thyroid surgery [30, 44–49]. Zedenius et al. performed 100 thyroidectomies with autotransplantation of parathyroid tissue and could not observe postoperative hypoparathyroidism. Their recommendation was to routinely perform parathyroid autotransplantation during thyroidectomy [50]. Another study by Lo et al. showed that the rate of postoperative hypoparathyroidism was significantly higher in patients after parathyroid autotransplantation (25%) than in patients without parathyroid autotransplantation (15%) [3]. Kihara et al. critically evaluated autotransplantation and are of the opinion that, if possible, all parathyroid glands should be preserved in situ [33]. Tartaglia et al. suggest that parathyroid autotransplantation has neither an effect on the rate of postoperative hypocalcemia nor on the rate of transient or permanent hypoparathyroidism [51].

In our analysis, 85 (23.8%) patients underwent thyroidectomy with selective parathyroid autotransplantation. Our data show that autotransplantation had a significantly negative effect on the postoperative function of the PGs. This can be explained by the fact that autotransplanted PGs start their endocrine activity only 2–3 weeks after surgery. According to our long-term follow-up results, the autotransplantation of PGs did not lead to a significantly higher rate of permanent hypoparathyroidism. It has to be emphasized, however, that in our patient with manifest permanent hypoparathyroidism, autotransplantation did not protect him from this irreversible complication. Moreover, it is impossible to verify whether the autotransplanted or the in situ preserved PG is responsible for the postoperative secretion of PTH after an autotransplantation. We recommend saving PGs in situ as accurately as possible, even if the gland is discolored or has only delicate vascular or soft tissue pedicle [14, 52]. If necessary, a thyroid capsule should be left behind either on the upper or the lower PG location in order to protect vascularization.

### Limitations

A limitation of this study is its retrospective analysis as well as the fact that only one surgeon’s outcome was analyzed. This single surgeon’s experience is not necessarily applicable to other surgeons.

## Conclusion

Even an experienced thyroid surgeon is not always able to identify all four PGs during thyroidectomy, and in some cases not even a single one. Generally, the identification of the PGs temporarily harms their secretory function and preservation is better than autotransplantation. In the long term, we could not confirm that visualization helps to prevent permanent hypoparathyroidism because of the limited number of patients included; nevertheless, a trend was observed. Therefore, all surgeons need to be aware that if they are not able to visualize PGs, even more effort and care are required to microsurgically search the resected thyroid specimen to detect any accidentally removed PGs in order to perform autotransplantation. It is more important not to overlook a PG than to identify a specific number of PGs.
